# Effects of 5-HT_2C_, 5-HT_1A_ receptor challenges and modafinil on the initiation and persistence of gambling behaviours

**DOI:** 10.1007/s00213-020-05496-x

**Published:** 2020-03-02

**Authors:** Trevor Humby, Georgia E. Smith, Rebecca Small, William Davies, Jenny Carter, Chloe A. Bentley, Catharine A. Winstanley, Robert D. Rogers, Lawrence S. Wilkinson

**Affiliations:** 1 Behavioral Genetics Group, Schools of Medicine and Psychology, Cardiff University, Cardiff, CF10 3AT UK; 2 MRC Centre for Neuropsychiatric Genetics and Genomics and Division of Psychological Medicine and Clinical Neurosciences, School of Medicine, Cardiff University, Cardiff, UK; 3 Neuroscience and Mental Health Research Institute, Cardiff University, Cardiff, UK; 4 Department of Psychology, University of British Columbia, Vancouver, Canada; 5 School of Psychology, Bangor University, Bangor, UK

**Keywords:** Gambling, Loss-chasing, 5-HT_1A_R, 5-HT_2C_R, 8-OH-DPAT, SB242084, Modafinil, Touchscreen

## Abstract

**Rationale:**

Problematic patterns of gambling are characterised by loss of control and persistent gambling often to recover losses. However, little is known about the mechanisms that mediate initial choices to begin gambling and then continue to gamble in the face of losing outcomes.

**Objectives:**

These experiments first assessed gambling and loss-chasing performance under different win/lose probabilities in C57Bl/6 mice, and then investigated the effects of antagonism of 5-HT_2C_R with SB242084, 5-HT_1A_R agonism with 8-OH-DPAT and modafinil, a putative cognitive enhancer.

**Results:**

As seen in humans and other species, mice demonstrated the expected patterns of behaviour as the odds for winning were altered increasing gambling and loss-chasing when winning was more likely. SB242084 decreased the likelihood to initially gamble, but had no effects on subsequent gambling choices in the face of repeated losses. In contrast, 8-OH-DPAT had no effects on choosing to gamble in the first place, but once started 8-OH-DPAT increased gambling choices in a dose-sensitive manner. Modafinil effects were different to the serotonergic drugs in both decreasing the propensity to initiate gambling and chase losses.

**Conclusions:**

We present evidence for dissociable effects of systemic drug administration on different aspects of gambling behaviour. These data extend and reinforce the importance of serotonergic mechanisms in mediating discrete components of gambling behaviour. They further demonstrate the ability of modafinil to reduce gambling behaviour. Our work using a novel mouse paradigm may be of utility in modelling the complex psychological and neurobiological underpinnings of gambling problems, including the analysis of genetic and environmental factors.

**Electronic supplementary material:**

The online version of this article (10.1007/s00213-020-05496-x) contains supplementary material, which is available to authorized users.

## Introduction

With increased access to opportunities to gamble in society, on the high street or via social media, more people are gambling these days than ever before. For most people, gambling is something that they can dip into occasionally and easily control, but for others, gambling can become a major problem affecting work and home life, personal relationships and financial security (Gainsbury 2015). Efforts to understand pathological gambling reveal a complex mix of genetic, familial and environmental risk factors, the former including gene variants that influence monoaminergic systems (Hodgins et al. 2011) and the latter socio-economic status, education, gender and accessibility to gambling platforms (van den Bos et al. 2013; Gainsbury 2015). Gambling problems are mediated by altered neuromodulation within mesolimbic reinforcement sites and altered neural responses to monetary rewards and gambling-related cues (Zack and Poulos 2004; Chase and Clark 2010; Balodis et al. 2012; Worhunsky et al. 2014). These dysfunctional brain responses may mediate the altered cognitions that promote gambling and, in vulnerable individuals, gambling problems (Campbell-Meiklejohn et al. 2008; Clark et al. 2009; Clark 2010). Current treatments include cognitive and/or behavioural therapies (Stea and Hodgins 2011) but also a variety of pharmacological options including SSRIs, mood stabilisers, atypical anti-psychotics and opioid antagonists (Grant et al. 2014). These therapies have uncertain efficacy, especially over the longer term, and there remains a need to better understand the neurobiology and risk factors associated with gambling behaviours and problematic gambling in order to identify more effective interventions and therapeutic targets (Potenza et al. 2013; Gainsbury 2015).

The progression from recreational gambling to pathological gambling is thought to be due to the loss of control and development of the characteristic loss-chasing behaviour (Lesieur 1979; Breen and Zuckerman 1999). Loss-chasing is the tendency to continue gambling or escalate stakes in the face of successive and accumulating losses (mainly financial) and may occur within a single session or over the longer term—both with the ultimate aim to ‘get even’ (Lesieur 1977, 1979). Between-session loss-chasing occurs, as the name suggests, over a longer period of time and may include multiple gambling sessions (Lesieur 1977). The majority of research has investigated within-session loss-chasing, where an individual attempts to recoup losses within a single session (Parke et al. 2016). Behavioural economic perspectives attribute loss-chasing to a convex function linking accumulating monetary losses to only diminishing marginal reductions in subjective value or utility, promoting further risk-seeking choices (Kahneman and Tversky 1979; Kahneman and Tversky 2000). However, in clinical populations, loss-chasing is markedly exaggerated and is associated with heightened trait impulsivity and erroneous false beliefs that support continued play (Breen and Zuckerman 1999). Loss-chasing is also associated with alcohol use and its disinhibitory effects (O'Connor and Dickerson 2003) and can be increased by alcohol consumption (Kyngdon and Dickerson 1999). These behavioural features make loss of control and consequent loss-chasing a key mechanism by which problem gambling produces its adverse financial, occupational and social consequences (Lesieur 1979).

There have been limited previous studies of the pharmacological basis of within-session loss-chasing. Using an experimental model in which human volunteers chose between gambling to recover accumulating (notional) losses by doubling their stakes or ‘quitting the chase’, Campbell-Meiklejohn and colleagues (Campbell-Meiklejohn et al. 2008, 2011) demonstrated that loss-chasing involves dissociable roles for monoamine systems, with serotonin activity modulating the persistence of chasing behaviour, but dopamine and, in particular, D_2_/D_3_ receptor (R) activity modulating the evaluation of losses worth chasing (Campbell-Meiklejohn et al. 2011, 2012; Rogers 2011). Exploiting the foraging preference of animals to access food promptly (Bateson and Kacelnik 1995; Kacelnik and Bateson 1996), Rogers et al. (2013) using rats modelled within-session loss-chasing as choices between immediate food access versus delayed access to food rewards (timeouts) over successive bad choice outcomes. With accumulating increases in delay to receiving reward as the penalty for continued gambling (and losing), whereas as quitting gambling would bring about reward sooner. Consistent with the human data (Campbell-Meiklejohn et al. 2011, 2012), the 5-HT_1A_R agonist 8-OH-DPAT and D_2_R antagonist eticlopride, diminished loss-chasing behaviour, increasing quitting at the first opportunity, whilst the D_1_R antagonist, SCH23390, had little effect (Rogers et al. 2013).

Both behavioural economic arguments and also the possibility that loss-chasing may be supported by behavioural momentum effects (defined as the persistence of gambling choices on the basis of previous winning outcomes/rewards in the same context) raise the possibility that different mechanisms mediate the commencement of gamble choices and their persistence. One hypothesis here, maybe the allocation of behavioural control of reinforcement learning balanced between model-based and mode-free systems, the former centred in prefrontal cortical regions and the latter in striatal regions of the brain. Complex or demanding situations may transition processing from the inflexible model-free system to the more flexible goal-directed model-based processing system (Daw et al. 2005; Russek et al. 2017; Kim et al. 2019). In terms of gambling and loss-chasing, then these systems could relate to the habitual nature of gambling (model-free), and the goal-directed actions underlying loss-chasing (model-based). Here, we investigate this dissociation using an adapted model of within-session loss-chasing for mice and use specific drug challenges to demonstrate novel pharmacological dissociations between the initial decision to gamble (to avoid timeouts and gain immediate palatable condensed milk rewards) and successive choices over pre-programmed sequences of losing outcomes.

Our data provide evidence consistent with the existence of neurobiologically distinct mechanisms underlying gambling and loss-chasing behaviour based on serotonergic signalling, via 5-HT_2C_R and 5-HT_1A_R mechanisms and also, for the first time in an animal model, the administration of the putative ‘cognition enhancer’ modafinil, which may have therapeutic potential in pathological gambling (Łabuzek et al. 2014; Pettorruso et al. 2014). In using mice in these studies, we also further demonstrate the tractability of this species in investigating complex behaviour and provide a new platform that can be used for the pharmacological or genetic investigation of gambling behaviours.

## Methods and materials

### Subjects

Two cohorts of male C57Bl/6OlaHsd mice (Envigo, UK) (*N* = 20/cohort), 3 months old at the start of the experiment, were housed in groups of four, in a vivarium (temperature: 21 ± 2 °C, humidity 50% ± 10) and a 12:12 light/dark cycle (lights on at 07:00 h). Food was available ad libitum; following 2 weeks of habituation and handling, the mice were placed on a home cage water restriction schedule of 2 h access/day, maintained for the duration of the experiment to motivate the animals to work in the task. Animals were treated in accordance with the Animal (Scientific Procedures) Act (United Kingdom, 1986).

### Apparatus

The gambling/loss-chasing task (G/LCT) was performed in four mouse touchscreen chambers (Campden Cognition, UK, and see Bussey et al. 2012), under the control of custom written software (ABET, Campden Cognition, UK). Touchscreens were occluded by black Perspex masks with three 70 mm square response apertures 20 mm from the grid base of the chamber and positioned equally across the width of the mask. In the opposite side of the chamber, 5 mm from the grid base, was a 20 mm square recess (25 mm deep) from which liquid reward was delivered. Infra-red beams were used to record motor activity.

### Procedure

**Fig. 1:**
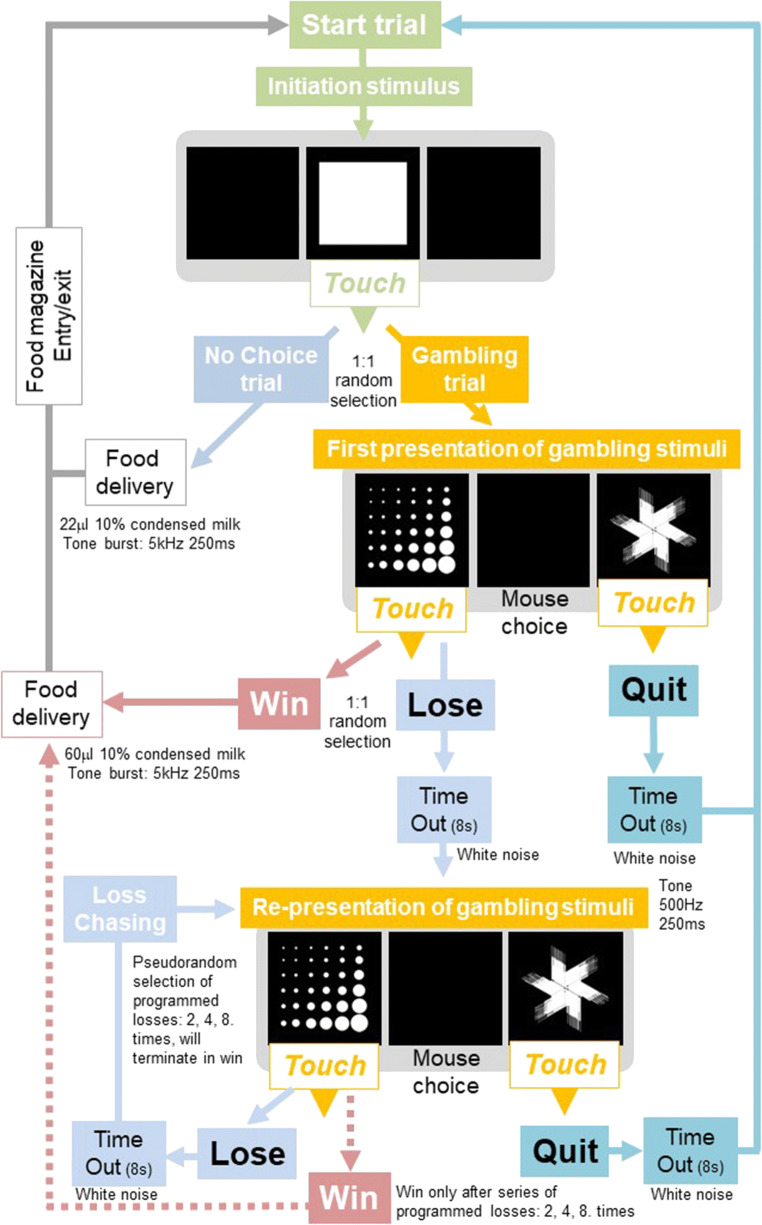
Schematic of the touchscreen gambling/loss-chasing task (G/LCT). The procedure was adapted from Rogers et al. (2013) and extended to a touchscreen platform in mice. A key modification to the current task was the introduction of procedures allowing a more systematic evaluation of loss-chasing behaviour over repeated programmed losses, allowing the opportunity for comparisons of loss-chasing to other components of gambling behaviour, such as the initial decision to quit or gamble. See main text and Supplementary Methods Figure 2 for further details.

Following 2 weeks home cage water restriction schedule, the mice were habituated to the liquid reward of 10% condensed milk (Nestle Ltd., UK) solution (Humby et al. 1999, 2005). Subjects were habituated to the touchscreen chambers for 3 days and then trained to make a response to the central touchscreen stimulus location to earn reward (see Supplementary Methods Figure 1. When mice showed stable responding (> 40 responses/session for two consecutive sessions), the G/LCT was commenced with an initial training baseline version of the task in which touch responses to the gamble stimulus produced winning and losing outcomes with equal probabilities, following which the full G/LCT schedule was implemented which included loss-chasing options (see Fig. 1). All sessions in the touchscreen chambers were performed in darkness.

The procedure for the G/LCT, adapted from Rogers et al. (2013), was modified for a touchscreen platform. Two basic types of trial were presented equally and randomly through each session: ‘no-choice’ and ‘choice’ (Fig. 1). In a no-choice trial, which was designed to maintain a high level of responding, the initiation stimulus was presented to the central aperture of the touchscreen, touching the stimulus resulted in a delivery of 22 μl of condensed milk reward in the food magazine. The other 50% of trials were choice trials in which ‘quit’ and ‘gamble’ choice stimuli (‘fan’ and ‘marbles’ patterns, respectively, see Fig. 1) were presented in the left and right apertures, following a response to the central initiation stimulus, with the location randomly assigned for each trial. Choosing the quit stimulus resulted in no reward and a time-out period of 8 s signalled by illumination of the houselight, following which a new trial of either no-choice or choice was initiated. Thus, quitting this initial choice prevented the gambling option and had the potential to bring about a new trial more quickly. In contrast to a quit response, selecting the gamble stimulus generated, pseudo-randomly, a reward delivery of 60 μl reward (a ‘win’) or commenced a time-out period of 8 s, signalled by illumination of the houselight (a ‘loss’). In a standard gamble session, the ratio of wins to losses for the initial decision to gamble was set at 1:1, but this initial gambling choice was also assessed in sessions with different win/lose ratios (see below).

**Fig. 2:**
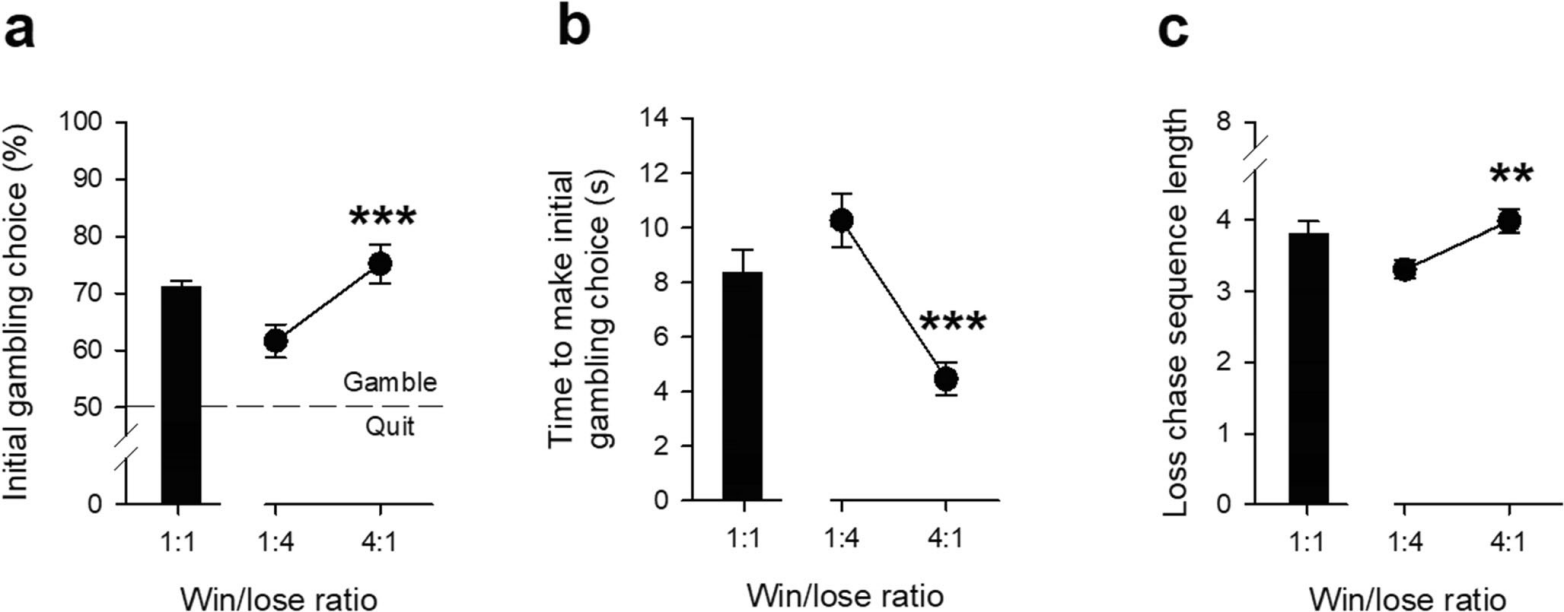
Effects of altering the odds of winning on initial gambling choice and loss-chasing behaviour in adult C57Bl/6 mice. When the odds of winning were high, the mice showed a greater initial preference to gamble (**a**), were quicker at making this choice (**b**), and were more likely to loss-chase (**c**). Data from the final day of testing at each schedule were used in the analysis. Data from sessions with a 1:1 win/lose ratio are shown for illustrative purposes and were not included in the statistical analysis. Data shows mean ± SE, for both mouse Cohorts combined (*N* = 40). ** denotes *p* < 0.01 and *** denotes *p* < 0.001 for the comparison between 4:1 and 1:4 win/ratio tests

Collection of the reward after a winning outcome started a new no-choice or choice trial, but in the case of a losing outcome, and following the time-out as above, loss-chasing was assessed by continuing the current trial and offering the opportunity for further gamble or quit choices. Here, the animals were exposed to a series of programmed series of 2, 4 or 8 or losing outcomes (presented in pseudorandom order) that were terminated, should an animal continue to gamble and lose for the entire loss-chasing sequence available, by a winning outcome (see Supplementary Methods Figure 2). This possibility was included in the task design to encourage loss-chasing behaviour in the mice. At any time during a loss-chase sequence, the mouse could make a quit response and terminate these losing runs, receiving a time-out prior to the start of a new trial. In every trial, the stimuli would remain on the screen until a response had been made and thus, there were no omitted trials or choices. Thus, the penalty for gambling and losing (rather than quitting a trial) was to increase the delay to the next available reward (like in the Rogers et al. 2013 task design). A session was terminated once the subject had completed 60 trials or 20 min had elapsed.

#### Task manipulations

##### Initial decision to gamble

To test the extent to which the initial decision to gamble was sensitive to the probability of winning outcomes, the mice were tested with counter-balanced sequences of sessions with win/lose ratios of 1:4, and 4:1 for the initial gamble. Mice underwent a sequence of 6 consecutive sessions at each ratio, with a block of 1:1 win/lose ratio baseline sessions in between each manipulation.

##### Loss-chasing

The extent to which the various win/lose ratios pertaining for the initial gamble influenced the degree of subsequent loss-chasing behaviour was also assessed.

##### Pharmacological manipulations

The sensitivity of initial gambling choice and loss-chasing behaviours to pharmacological challenge were assessed in baseline 1:1 win/lose ratio sessions. This win/lose ratio was selected as it was equivalent to the rat task on which this study was based (Rogers et al. 2013), but also as it permitted both increases and decreases in gambling choices allowing for a non-biased hypothesis for the possible effects of the different drugs used. 5-HT_2C_R and 5-HT_1A_R were manipulated using SB242084 HCl (0, 0.1, 1, 5 mg/kg) and 8-OH-DPAT HCl (0, 0.03, 0.06, 0.1 mg/kg) (Cohort 1, *N* = 20 mice), respectively. Each of these drugs showing high selectivity for their target receptors (pKi = 9.0 and, pKi = 8.8, for SB242084 and 8-OH-DPAT, respectively (Kennett et al. 1997; Assié and Koek 2000). The effects of modafinil (0, 32, 64 mg/kg) were assessed in a separate group of mice (Cohort 2, N = 20) (all Tocris, UK). Doses of SB242084 (s.c., administered immediately prior to test), 8-OH-DPAT (i.p., administered 20 min prior to test) and modafinil (i.p., 30 min before test) were based on previous studies (Humby et al. 2013; Fernandes et al. 2013; Rogers et al. 2013). Drugs were prepared fresh in physiological saline each day. Each treatment was given using a Latin-square design, with at least 4 days between each dose; mice were not tested between the drug administration sessions.

### Statistics

Main measures for the G/LCT were the % initial gambling choice, mean length of a loss-chase sequence, latency to make an initial gambling choice, proportion loss-chases ended by a quit response, number of trials started, latency to initiate a trial, latency to collect the reward and the numbers of food magazine entries, beam-breaks and touches to empty apertures of the touchscreen (blank touches). Scores calculated as percentages were arcsine transformed, and latencies were square root transformed prior to analysis. Data were analysed using SPSS (V.20, IBM Inc., USA) and were first assessed for normality, and then subjected to *t* test or ANOVA, if appropriate or equivalent non-parametric analyses. The comparison of performance with high (4:1) or low (1:4) win/lose ratios was analysed by paired two-tailed *t* tests or Wilcoxon signed-rank test. These analyses were performed with the two mouse cohorts combined, but also for comparisons between the two groups (Supplementary Results Figures 4 and 5). The effects of SB242084, 8-OH-DPAT and modafinil were analysed by separate within-subject ANOVAs or Friedman tests with a factor of DOSE (vehicle, 0.1, 1, 5 mg/kg, vehicle, 0.03, 0.06, 0.1 mg/kg, and vehicle, 32 and 64 mg/kg, for each drug, respectively). If significant, then post hoc pairwise comparisons were performed using Bonferroni or Wilcoxon signed-rank test, respectively, and adjusted for multiple comparisons. Criterion level of significance was set at the 0.05 level. All data are shown as mean ± standard error of the mean (S.E.M.).

## Results

Prior to testing, all 40 mice in the experiment demonstrated significant preference for the condensed milk reward over water (c. 80% preference, see Supplementary Results Figure 1), consistent with previous groups of mice (Humby et al. 1999, 2005, 2013). Following habituation and shaping (see Supplementary Results Figure 2), the mice achieved reliable and stable performance in the G/LCT within 20 sessions (mean 19.73 ± 0.47 sessions).

### C57Bl/6 mice will gamble and chase losses for food reward

**Fig. 3:**
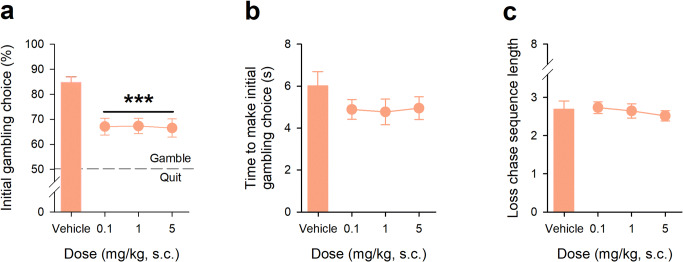
Effects of 5-HT_2C_R antagonist SB242084 on initial gambling choice and loss-chasing behaviour in adult C57Bl/6 mice. At all doses used, SB242084 reduced the initial choice to gamble (**a**). There were no effects of SB242084 at any dose on the time taken to make the initial choice to gamble (**b**), or on the number of loss-chases made by the mice (**c**). For each of these sessions, the win/lose ratio was 1:1. Data shows mean ± SE, mice from Cohort 1 only (*N* = 20). *** denotes *p* < 0.001 for the comparison with vehicle treatment

**Fig. 4:**
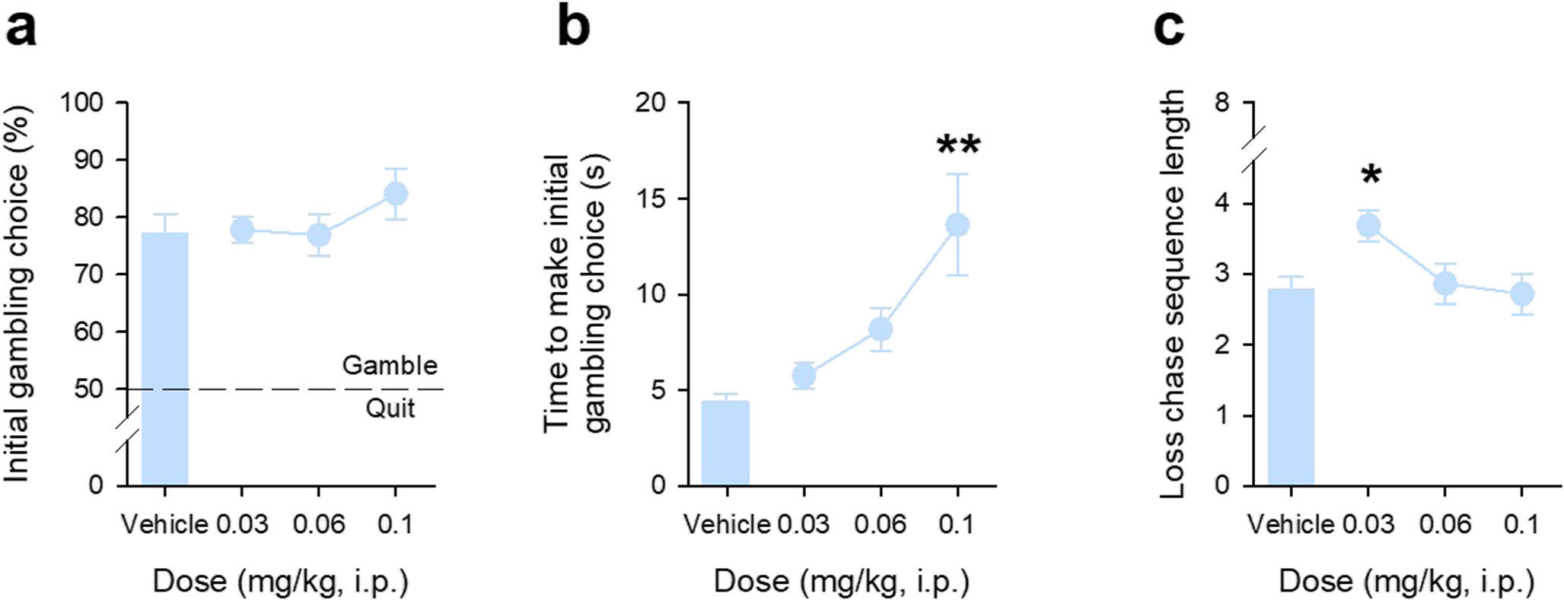
Effects of the 5-HT_1A_R agonist 8-OH-DPAT on initial gambling and loss-chasing behaviour in adult C57Bl/6Ola mice. 8-OH-DPAT did not significantly affect the initial choice of the mice to gamble (**a**), but increased the time taken to make this choice, although this was only at the 0.1 mg/kg dose used (**b**). Loss-chasing was influenced by 8-OH-DPAT, with a significant increase from vehicle that was specific to the lowest 0.03 mg/kg dose of the drug (**c**). For each of these sessions, the win/lose ratio was 1:1. Data shows mean ± SE, mice from Cohort 1 only (*N* = 20). * denotes *p* < 0.05 and ** *p* < 0.01 for comparison with vehicle treatment, respectively

**Fig. 5:**
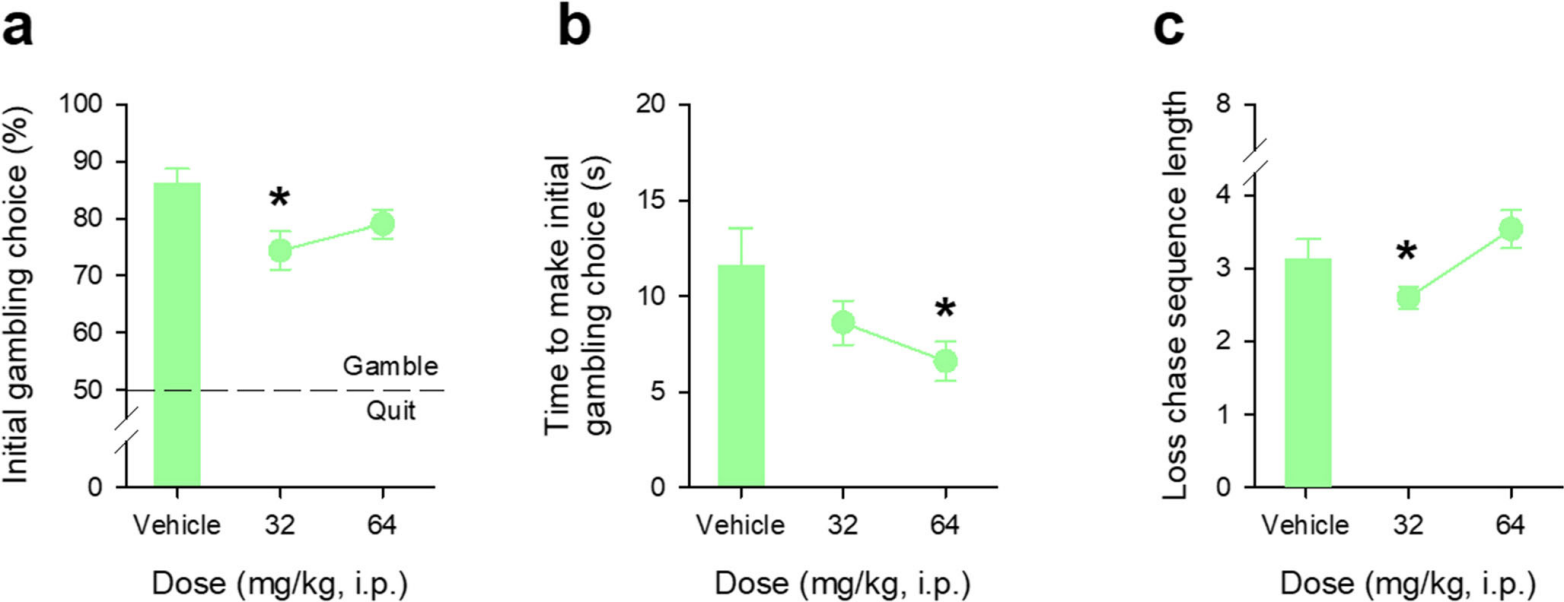
Effects of modafinil on gambling and loss-chasing behaviour in adult C57Bl/6 mice. Modafinil reduced the initial decision to gamble by the mice at the 32 mg/kg dose with no significant effects of the higher 64 mg/kg dose (**a**). Modafinil reduced the time taken to make the decision to gamble, effects that reached significance at the 64 mg/kg dose but not 32 mk/kg dose (**b**). Loss-chasing was also influenced by modafinil, with a significant reduction in behaviour from vehicle at 32 mg/kg but not 64 mg/kg (**c**). For each of these sessions, the win/lose ratio was 1:1. Data shows mean ± SE, mice from Cohort 2 (*N* = 20). * denotes *p* < 0.05 for the comparison with vehicle treatment

Having learned to touch the screen for reward and shown consistent and stable performance in the baseline version of the G/LCT with 1:1 win/lose ratio, the mice were moved on to the task configuration that included a loss-chasing component. Under the baseline 1:1 win/lose ratio, for the initial decision to gamble or quit the animals made more gamble than quit choices (c.70% gamble choice, see Fig. 2a). As anticipated, the initial decision to gamble was also sensitive to the probability of gaining reward with more gamble selections (Fig. 2a, *t*_39_ = 5.57, *p* = 0.001, *d* = 0.33) and quicker responses (Fig. 2b, *Z*_39_ = 5.26, *p* = 0.001, *d* = 0.36) in sessions with high win/lose ratios than low win/lose ratios. These mice also demonstrated loss-chasing behaviour whereby following the initial choice to gamble and a loss the animals continued to gamble for reward despite suffering repeated further losses and associated time-out penalties. As shown in Fig. 2c, the mean length of consecutive loss-chases before quitting, under the baseline 1:1 win/lose ratio condition, was ~ 4 losses (the mice rarely persisted in chasing all 8 losses available, see Supplementary Results Figure 3a). Loss-chasing behaviour was sensitive to the prevailing win/lose ratio of the initial gamble with animals engaging in longer chases in sessions with a high win/lose ratio than in sessions with a low win/lose ratio (Fig. 2c, *Z*_39_ = 2.81, *p* = 0.005, *d* = 0.28). As two cohorts of mice were used in these studies, we have also performed between-cohort comparisons on these data, which demonstrated non-significant results (*p* > 0.05 for all analyses (Supplementary Results Figure 4). We also assessed the comparison between cohorts using a Bayesian approach (JASP V0.11, Netherlands) which demonstrated very weak effects for differences between the two cohorts of mice across all the measures (BF_10_ < 1, odds ratio relative to the null hypothesis). Therefore, both groups of mice showed equivalent responses as the win/lose ratio was altered. However, we did notice that there were some changes in performance with the baseline 1:1 win/lose ratio between the first time of test and on completion of the win/lose ratio assessments (Supplementary Results Figure 5). We assessed these differences and found that the propensity to gamble had significantly increased, and the loss-chase sequence length reduced for both cohorts of mice; however, we also found that these differences remained constant and unchanging through evaluation of the effects of the drug challenges (Supplementary Results Figure 5, comparison with vehicle treatment).

### 5-HT_2C_R antagonism with SB242084 reduces the propensity to gamble without influencing loss-chasing

The effects of the 5-HT_2C_R antagonist SB242084 on the initial decision to gamble and subsequently chase losses are shown in Fig. 3. The effects of the drug showed dissociations between these different phases of behaviour. SB242084 at doses of 0.1, 1 and 5 mg/kg reduced the propensity to make an initial gamble; that is, the animals showed a clear reduction in gambling responses and, therefore, a corresponding increase in quitting, under drug (Fig. 3a, main effect of DOSE, *F*_3,57_ = 9.27, *p* = 0.001, *η*^2^ = 0.99). The effects of SB242084 on whether to gamble or quit occurred in the absence of effects on the speed of responding (Fig. 3b, main effect of DOSE, *F*_3,57_ = 2.24, *p* = 0.09, *η*^2^ = 0.54). The effects of SB242084 on the initial decision to gamble did not extend to subsequent loss-chasing, where behaviour was insensitive to drug, insofar as there were no effects at any dose on the persistence of gambling in the face of losing runs (Fig. 3c, main effect of DOSE, *F*_3,57_ = 0.29, *p* = 0.82, *η*^2^ = 0.10). The dissociations in behaviour were highly specific and occurred in the absence of drug-induced changes in general components of behaviour for example, the number of responses/session, latency to start a trial, locomotor activity and food magazine entries, although there was a tendency for an increased reward collection latency with the 5 mg/kg dose (Supplementary Results Figure 3).

### 5-HT_1A_R agonism with 8-OH-DPAT has no effects on the initial decision to gamble but increases loss-chasing

In contrast to SB242084, 8-OH-DPAT had no statistically significant effects on the initial choice to gamble (Fig. 4a). Note that the main effect of DOSE was close to significance (*F*_3,57_ = 2.86, *p* = 0.06, *η*^2^ = 0.58), because of elevated rates of gamble responses at the highest dose used, of 0.1 mg/kg; however, this dose was also associated with major disruptions in general components of behaviour, including marked slowing of choice responding (Fig. 4b, main effect of DOSE, *F*_3,57_ = 11.20, *p* = 0.001, *η*^2^ = 0.99) and a decline in the overall number of trials started in a session from ~ 60 under vehicle conditions to ~ 40 (see Supplementary Results Figure 4). There was evidence of specific effects of 8-OH-DPAT on loss-chasing behaviour. These effects were manifest as an increase in consecutive loss-chases at the lowest, 0.03 mg/kg, dose of drug (Fig. 4c, main effect of DOSE, *F*_3,57_ = 4.48, *p* = 0.01, *η*^2^ = 0.86, confirmed by post hoc comparison: *p* < 0.05 for 0.03 mg/kg dose to all other doses) in the absence of generalised effects on other behavioural measures (Supplementary Results Figure 4).

### Modafinil reduces both the propensity to gamble and loss-chasing behaviour

Modafinil reduced initial choices to gamble, thereby increasing quitting behaviour (Fig. 5a, main effect of DOSE, *F*_2,38_ = 4.28, *p* = 0.02, *η*^2^ = 0.18); post hoc comparisons showing that choices to start gambling at the 32 mg/kg dose were significantly diminished compared to vehicle (*p* = 0.035), but not with the 64 mg/kg dose (*p* = 0.84). The effects of the 32 mg/kg dose of modafinil in reducing gambling choices were not associated with any marked effects on the speed of responding; however, the 64 mg/kg dose reduced speed of responding (Fig. 5b, main effect of DOSE, *F*_2,38_ = 4.63, *p* = 0.02, *η*^2^ = 0.18; comparisons of vehicle and 32 mg/kg: *p* = 0.80, and vehicle and 64 mg/kg *p* = 0.045). Modafinil reduced the extent of subsequent loss-chasing but again only at the lower 32 mg/kg dose (Fig. 5c, main effect of DOSE, *F*_2,38_ = 7.14, *p* = 0.002, *η*^2^ = 0.27, post hoc comparisons of 32 mg/kg dose with vehicle and 64 mg/kg of *p* = 0.03 and *p* = 0.001, respectively). Neither dose was associated with any marked change in general features of behaviour save a slight tendency for the overall number of trials started to increase at the highest dose (Supplementary Results Figure 5).

## Discussion

We have used a novel touchscreen-based paradigm in mice to investigate gambling behaviours. We established that hungry mice will gamble and loss-chase to achieve food reward, with the possible penalty of losing as increased delay to the next available reward. The mice were sensitive to the relative probabilities of winning/losing outcomes, as seen in humans and other species mice, by increased gambling and loss-chasing when winning was more likely or quitting gambling when winning was less likely. We found evidence for the existence of dissociable effects of serotonergic drugs and modafinil on different aspects of gambling behaviour. Antagonism of 5-HT_2C_R with SB242084, whilst decreasing the likelihood of C57Bl/6 mice to make an initial gambling choice, had no effects on the persistence of subsequent loss-chasing behaviours. In contrast, 5-HT_1A_R agonism, with low dose 8-OH-DPAT, had no effects on the likelihood of subjects choosing to initiate gambling, but once they had gambled tended to increase the subsequent loss-chasing behaviours. Modafinil at a dose of 32 mg/kg decreased both the propensity to engage in gambling and reduced loss-chasing. Our data are consistent with the suggestion that serotonin receptor systems may play dissociable roles in starting and then sustaining gambling responses in the face of continued losing outcomes (Campbell-Meiklejohn et al. 2011; Rogers et al. 2013); additionally, they indicate the efficacy of modafinil, a putative cognitive enhancer, to modify gambling behaviours.

Human studies have attempted to parse components of gambling behaviours and there is evidence for the existence of dissociable neural systems and pharmacological substrates underlying, for example, the decision to chase losses or quit (Campbell-Meiklejohn et al. 2008, 2011 and 2012). However, to our knowledge, there have been no explicit comparisons of the pharmacological mechanisms of initial decisions to gamble/quit and subsequent loss-chasing behaviour in either un-drugged or drug treated subjects. Behavioural economic perspectives, such as Prospect Theory (Kahneman and Tversky 1979; Kahneman and Tversky 2000), posit that within-session loss-chasing behaviour persists because in the case of typical human gambling behaviour, accumulating monetary losses generate only diminishing marginal reductions in subjective value (relative to gamblers’ current purse), prompting the persistence of risk-seeking choices (Campbell-Meiklejohn et al. 2008). According to this view, initial decisions to gamble are distinguished by the potential for greater reductions in subjective value compared to subsequent decisions to continue gambling. Additionally, loss-chasing may also be contributed to by a form of behavioural momentum, manifest as the persistence of choices previously but not currently rewarded in the same context (Kahneman and Tversky 1979; Nevin 2002). Irrespective of these theoretical speculations regarding the psychological underpinnings of gambling behaviours, the pharmacological data presented here suggest that 5-HT_2C_R and 5-HT_1A_R activity modulate risky choices to varying extents between the initial decision and subsequent loss-chasing.

Our findings add to the increasing evidence for serotonergic neurotransmitter influences on gambling behaviours as demonstrated in both human and animal model studies (Zeeb et al. 2009; Campbell-Meiklejohn et al. 2011; Rogers et al. 2013). A novel finding was the efficacy of SB242084 to alter gambling behaviour by reducing the propensity to make an initial gamble (increased quitting) with no effects on loss-chasing. Quitting the initial gambling choice, and receiving a time-out, could be interpreted as choosing the safer option which was more guaranteed to provide reward quicker than electing to gamble which induced further delays. Changes in 5-HT_2C_R function, specifically hypersensitivity, have been suggested to occur in pathological gamblers (Conversano et al. 2012). Hence, the data with SB242084 are consistent with human work; however, the precise psychological and brain mechanisms by which 5-HT_2C_R antagonism impacts on dissociable aspects of gambling behaviour are unknown but could be centred in the prefrontal cortex where these receptors are expressed (Pompeiano et al. 1994). One possible, hypothesis may be the involvement of cortical 5-HT_2C_R in top down processing and control of model-based and striatal model-free mechanisms for behavioural choice (Daw et al. 2005; Russek et al. 2017; Kim et al. 2019). In the current task, 5-HT_2C_R antagonism could influence the dissociation between these systems, via modulating striatal dopamine (Seymour et al. 2012) in the drive to move from initially gambling (and losing) to loss-chasing to pursue a potential win. However, previous studies have demonstrated the effects of SB242084 on response control and selection are complex, with evidence for both decreased (Humby et al. 2013) and increased impulsivity in control of choices (Winstanley et al. 2004; Fletcher et al. 2007; Robinson et al. 2008; Paterson et al. 2012). Acute activation of 5-HT_2C_R inhibits feeding (Clifton and Kennett 2006), and various 5-HT_2C_R agonists are currently registered to treat obesity (Palacios et al. 2017). SB242084 will reduce the anorectic effects of d-fenfluramine, mCPP or novelty-induced hypophagia, but does not affect food intake when administered alone (Vickers et al. 2001; Hewitt et al. 2002; Nahata et al. 2013). Thus, the findings observed here are unlikely to be a due to effects of SB242084 on feeding mechanisms or drive to achieve reward. Nonetheless, 5-HT_2C_Rs are expressed in brain circuitries with established roles in mediating response control and responses to reward (Barnes and Sharp 1999; Chagraoui et al. 2016) and further investigation of these psychological functions in the context of explaining the putative specificity of 5-HT_2C_R blockade on gambling behaviours is warranted.

In previous work using rat models, 8-OH-DPAT has been shown to reduce loss-chasing behaviour (Rogers et al. 2013). Using the same dose range in the current mouse task, we observed no significant effects on the initial decision to gamble but instead an increase in loss-chasing that was confined to the lowest 0.03 mg/kg dose consistent with a pre-synaptic effect of 5-HT_1A_R agonism (i.e. reduced 5-HT release, Sharp et al. 1989). There are several possible reasons for the contrasting effects of 8-OH-DPAT on loss-chasing seen here and in Rogers et al. (2013) work, which may include species differences but also a task difference which may be interacting with reduced 5-HT release. Specifically, in the present study, the subjects could endure up to 8 successive losses with the possibility of reward at the end, whereas in the rat task loss-chasing was programmed to curtail following only 2 extra gambles followed by a punishment (Rogers et al. 2013). This difference may have increased the propensity to loss-chase by the mice as a result of increased behavioural momentum, linked to the fact that increased persistence could result in reward, or as an effort to overcome the potentially greater number of negative outcomes (represented as accumulating delay to the next available reward) present in our mouse task (Nevin 2002). A further hypothesis is related to the involvement of 5-HT mechanisms in mediating feeding (Palacios et al. 2017), where it could be that increased loss-chasing reflected a drive to seek food by continued gambling. Although 8-OH-DPAT treatment increases feeding in non-food deprived rodents (Shepherd and Rogers 1990; Ebenezer and Surujbally 2007), it may lead to decreased or no changes in consumption of palatable foodstuffs (e.g. condensed milk rewards, as used in the current study) at doses comparable with those used here (Dourish et al. 1988; Swiergiel & Dunn 2000; Ebenezer and Tite 2003), thus a feeding-related hypothesis for our findings seems unlikely.

There have been suggestions that putative ‘cognition enhancers’ such as modafinil may be of use in pathological gambling (Łabuzek et al. 2014; Pettorruso et al. 2014). We tested the effects of modafinil in our model and found dose-sensitive reductions in both initial gambling choice and subsequent loss-chasing behaviour. In humans, modafinil has complex effects on gambling behaviours, with dissociable effects in problem gamblers that are dependent on high/low impulsivity traits, such as reducing the desire to gamble, the salience of gambling stimuli (such as gambling-related words) and critically, making risky choices (Zack and Poulos 2004; Smart et al. 2013). In the present work, we did not divide our experimental group into subgroups based on measures of individual impulsivity but found consistent effects of modafinil in reducing gambling choices. The psychological substrates of these effects from the data to hand remain unknown but the most prominent and robust effect of modafinil seems to be on general arousal and attention, rather than any specific cognitive process, via cortical mechanisms (Scoriels et al. 2013; Smart et al. 2013). On that basis, it might be speculated that modafinil could impact on multiple facets of loss-chasing behaviour, including, as we demonstrate here, the control of impulses to gamble in the first place to the control of persistent gambling in the face of repeated losses.

The precise neurobiological mechanism(s) of action of modafinil are, at present, also obscure. Modafinil does not appear to act as a monoamine releaser, as is the case for amphetamine-based stimulants, but has diverse effects on multiple neurotransmitter and modulatory systems encompassing, α-adrenoreceptors, GABA, orexin, dopamine and glutamate systems (Ballon and Feifel 2006), many of which have been implicated in gambling, and underlying psychologies mediating risk and decision making (Potenza 2013). For example, noradrenaline and orexins can affect motivation, arousal and decision-making (Chen et al. 2015; Quintero Garzola 2019) and decreased sensitivity of α2-adrenoreceptors has been reported in pathological gamblers (Quintero Garzola 2019), furthermore, clonidine, an α2-adrenoreceptor agonist decreased risky choice by decreasing reward sensitivity responding in a rat risk-taking task (Montes et al. 2015). Infusions of GABAA and GABAB receptor agonists into the prelimbic or infralimbic regions of the frontal cortex affect decision-making in a rat gambling task (Zeeb et al. 2015), and ketamine and MK801 NMDA receptor antagonists, affecting glutamate transmission, also influence decision making in probabilistic discounting tasks (Yates 2019). Clearly, more work is required to specify the precise brain mechanisms mediating the effects of modafinil on gambling behaviours.

In conclusion, we have developed a novel touchscreen assay to examine dissociable aspects of within-session gambling behaviour in mice. Mice demonstrated the expected patterns of behaviour when the odds for winning were altered resembling gambling behaviours seen in humans and other model species. With mice still ahead of rats as the main tractable genetic model for behavioural and neurobiological research (Muñoz-Fuentes et al. 2018), there is still a need for the development of novel methods of evaluating ‘normal’ mouse behaviour as a means to understand atypical patterns of behaviour following genetic or pharmacological manipulation. Thus, developing tasks such as the G/LCT will enable researchers to investigate a range of mechanisms related to pathological gambling (and response control) using mice as a model organism. We probed neurobiological mechanisms using pharmacological manipulations of the serotonergic system, specifically 5-HT_2C_ and 5-HT_1A_ receptors, and the putative cognition enhancer modafinil. The drugs had different effects on gambling behaviours providing evidence consistent with there being different brain mechanisms and systems operating to influence an initial decision to gamble and subsequent decisions to continue gambling in the face of repeated losses. Loss-chasing behaviour is a key component of problem gambling; hence, this work may have utility in modelling the complex psychological and neurobiological underpinnings of pathological gambling.

## Electronic supplementary material

ESM 1 (DOCX 1633 kb)
