# Supplementary material for: Effects of 5-HT2C, 5-HT1A receptor challenges and modafinil on the initiation and persistence of gambling behaviours
Source: Psychopharmacology (Berl). 2020 Mar 2;237(6):1745–56. doi: 10.1007/s00213-020-05496-x (PMC7239826; doi:10.1007/s00213-020-05496-x)
Supplement: Supplementary file 1 — (DOCX 1633 kb) [file 213_2020_5496_MOESM1_ESM.docx]

Effects of 5-HT_2C_, 5-HT_1A_ receptor challenges and modafinil on the initiation and persistence of gambling behaviours

Trevor Humby^1^*, Georgia E Smith^1^, Rebecca Small^1^, William Davies^1^, Jenny Carter^1^, Chloe A Bentley^1^, Catharine A Winstanley^2^, Robert D. Rogers^3^, Lawrence S. Wilkinson^1*^

^1^Behavioural Genetics Group, Schools of Medicine and Psychology, Cardiff University, UK; MRC Centre for Neuropsychiatric Genetics and Genomics and Division of Psychological Medicine and Clinical Neurosciences, School of Medicine, Cardiff University, UK; Neuroscience and Mental Health Research Institute, Cardiff University, UK.

^2^Department of Psychology, University of British Columbia, Canada

^3^School of Psychology, Bangor University, UK

*to whom correspondence should be addressed:

Dr Trevor Humby, e-mail: humbyt@cardiff.ac.uk

Professor Lawrence Wilkinson e-mail: wilkinsonl@cardiff.ac.uk

**Supplementary Information**

| - Supplementary Methods Figure 1 - Supplementary Methods Figure 2 - Supplementary Results Figure 1 - Supplementary Results Figure 2 - Supplementary Results Figure 3 | - Supplementary Results Figure 4 - Supplementary Results Figure 5 - Supplementary Results Figure 6 - Supplementary Results Figure 7 - Supplementary Results Figure 8 |
| --- | --- |

| 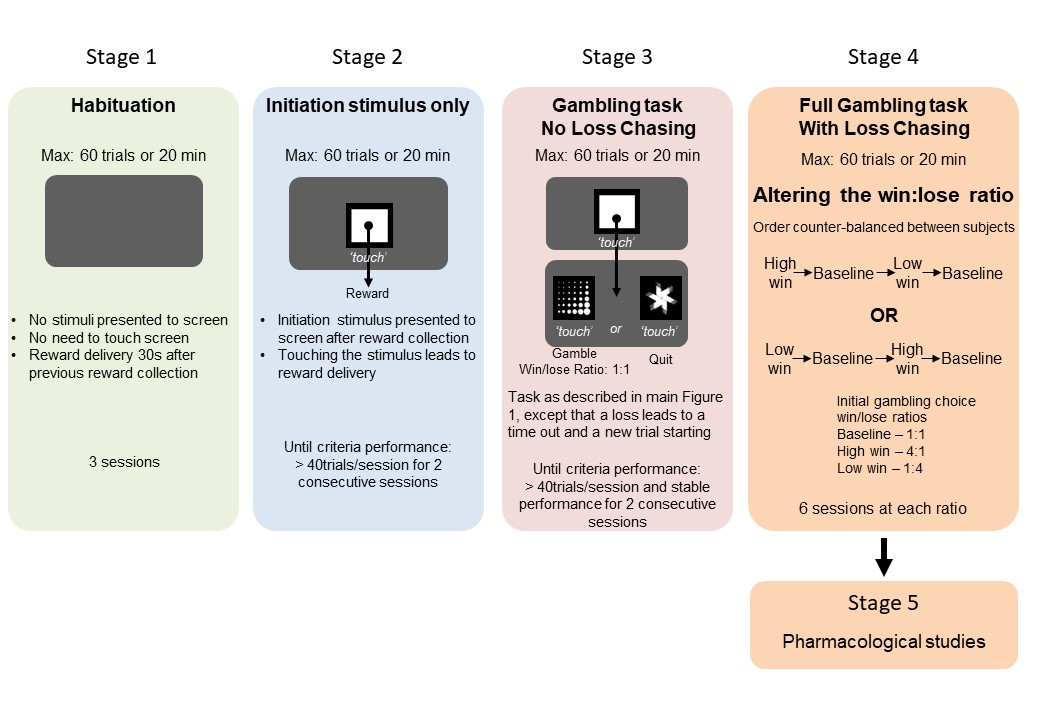 |
| --- |
| **Supplementary Methods Figure 1:** Experimental protocol. Following habituation to the food reward, the mice were habituated to the touchscreen chambers (Stage 1, 3 sessions) and learnt that the condensed milk reward was available in the food magazine. During these sessions no images were presented on the touchscreens. Following habituation, the mice were trained to touch the screens in response to presentation of the ‘initiation stimulus’ (Stage 2), a white square presented in the central aperture of the Perspex mask in from of the touchscreen monitor. When mice showed stable responding to the initiation stimulus, i.e. >40 trials/session for two consecutive sessions, the gambling/loss-chasing task (G/LCT) task was commenced with initial training in a non-loss-chasing version of the task (Stage 3, and see main text Figure 1). On 50% of trials, touching the initiation stimulus led to the presentation of the gamble or quit stimuli, pseudorandomly to the left or right stimuli apertures. In these sessions the win/lose ratio was 1:1, such that a gamble choice would lead to a loss or win 50% of the times each. A quit choice led to a period of time-out, signalled by illumination of the houselight. When the mice were performing >40 trials/session for two consecutive sessions and their gambling performance was stable, the full G/LCT schedule (Stage 4), including loss-chasing options, was implemented and performance assessed with different win/lose ratios. The order of these sessions was counter-balanced between mice. On completion of these task manipulations, a win/lose ratio of 1:1 was reinstated for 3 sessions, before performance of the mice was assessed under the different pharmacological challenges (Stage 5). There was a single session/day for each subject, and all sessions in the touchscreen chambers were performed with the houselight off. |

| 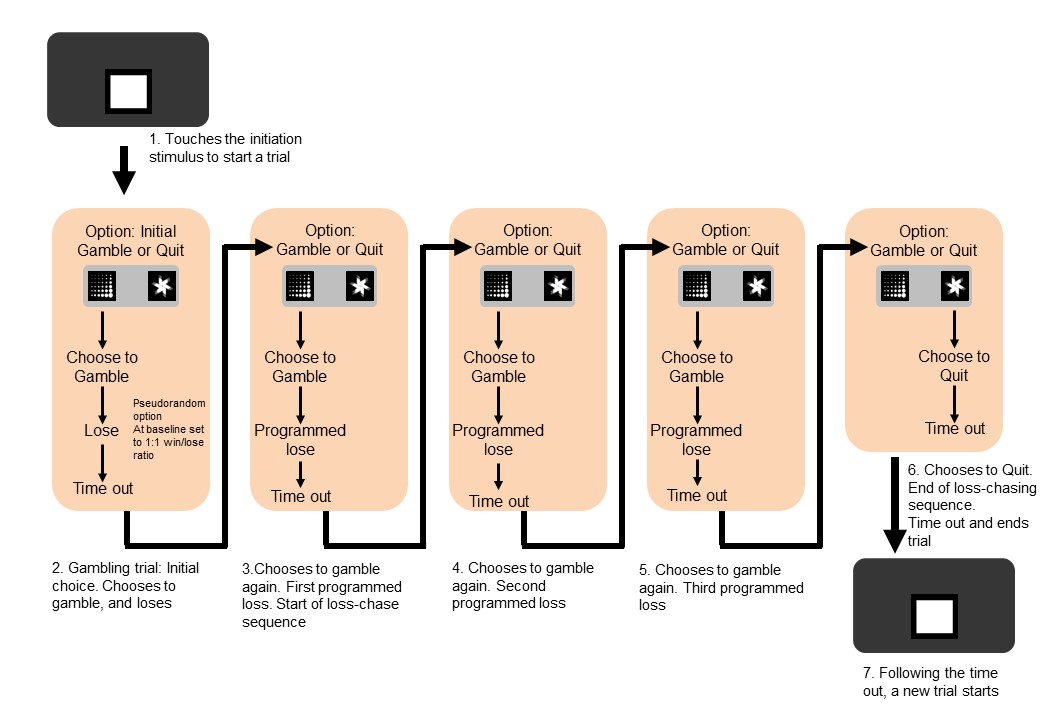 |
| --- |
| **Supplementary Methods Figure 2:** Schematic showing how a loss chasing sequence operated. The example shows a loss-chasing sequence consisting of a ‘mouse’ initially gambling and losing, then choosing to gamble for a further 3 times, before finally choosing to quit on the 5^th^ gamble/quit stimuli presentation. An 8s time-out, signalled by illumination of the houselight occurred following each loss, and the quit choice (which also led to the end of the trial). Only the initial gambling choice was subject to the prevailing win/lose ratio, all further gambling choices were programmed to be losses. The example shows how a loss-chase sequence could be terminated by quitting, but the length of loss-chasing sequences were pseudo-randomly determined to be of different programmed lengths (2, 4 or 8) that would end in a win and presentation of reward (See main Figure 1). However, on average the majority of loss-chases were terminated by the mice quitting before the end of the programmed sequence (Supplementary Results Figures 3e, 4a, 6a, 7a and 8a). |

| **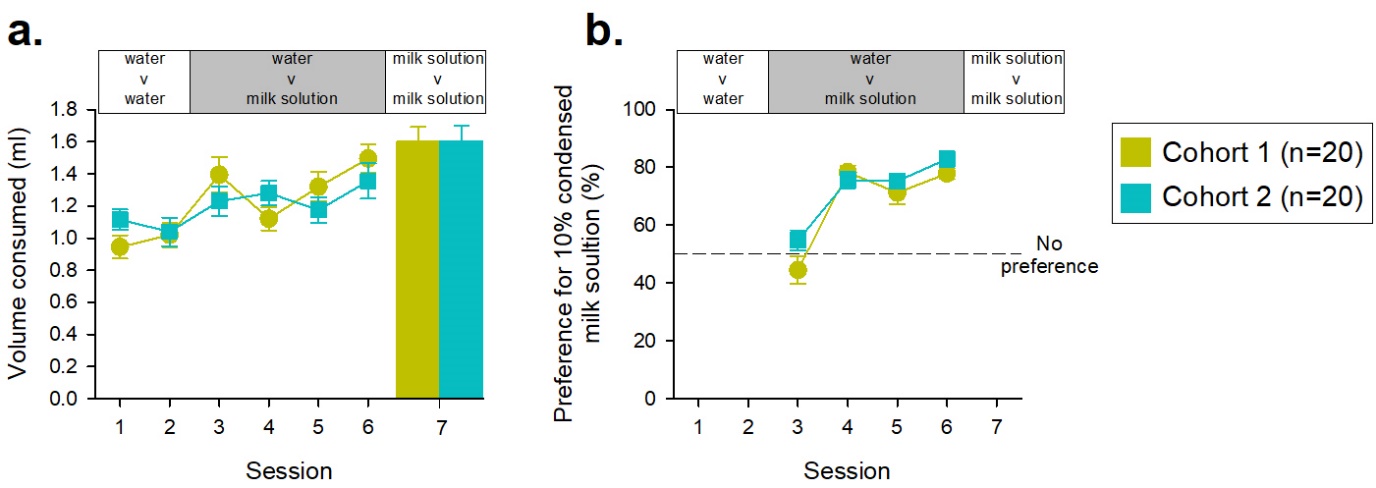** |
| --- |
| **Supplementary Results Figure 1:** Reward habituation and preference testing. The reinforcer preference test was carried out over a seven-day period (10 minute session/day) following previously published methods (Humby et al., 1999 and 2005). Mice were individually put into small cages (285 x 130 x 120mm), in which two containers (max vol.= 2ml) were placed to the rear of each cage. In the first two sessions, both containers contained tap water, and over the next four sessions, one of the containers was filled with the 10% condensed milk reinforcer solution. The locations of the two containers were pseudorandomly switched between sessions. On the final day of testing both containers were filled with the milk solution, to make sure that all mice had experienced the reward before subsequent testing. The containers were weighed prior to, and immediately after, testing to determine the consumption of each liquid as well as the preference for condensed milk. Reinforcer preference was defined as the amount of condensed milk consumed, as a percentage of the total amount of liquid consumed. Analysis by ANOVA found that there was a significant and very strong within-subject effect of SESSION (F_6,228_=15.26, p=0.001, η^2^=0.18,), demonstrating that all the mice increased consumption as the test progressed (a). Furthermore, the mice also demonstrated increasing preference (and reduced neophobia) for the milk reinforcer solution (b, effect of SESSION (F_6,228_=37.74, p=0.001, η^2^=0.49) achieving preferences >80% by the final day of preference testing. This level of reinforcer preference may reflect the fact that some of the mice may have consumed all the milk solution available and switched to drinking water. However, all the subjects consumed milk solution in the 7^th^ session, therefore all the mice had good experience of the reward before testing sessions in the touch-screen chambers started. There were no differences between the two cohorts of mice for either the volumes of solution consumed or reinforcer preference (main effect of COHORT, F_1,38_=2.70, p=0.11 and F_1,38_=3.10, p=0.09, η^2^=0.08, respectively), confirmed by Bayesian analysis. Data shows mean±SE, N=20/cohort.  1. Humby T, Laird FM, Davies W, Wilkinson LS. (1999) Visuospatial attentional functioning in mice: interactions between cholinergic manipulations and genotype. Eur J Neurosci. 11:2813-2823.  2. Humby T, Wilkinson L, Dawson G (2005) Assaying aspects of attention and impulse control in mice using the 5-choice serial reaction time task. Curr Protoc Neurosci. Ch. 8:Unit 8.5H |

| 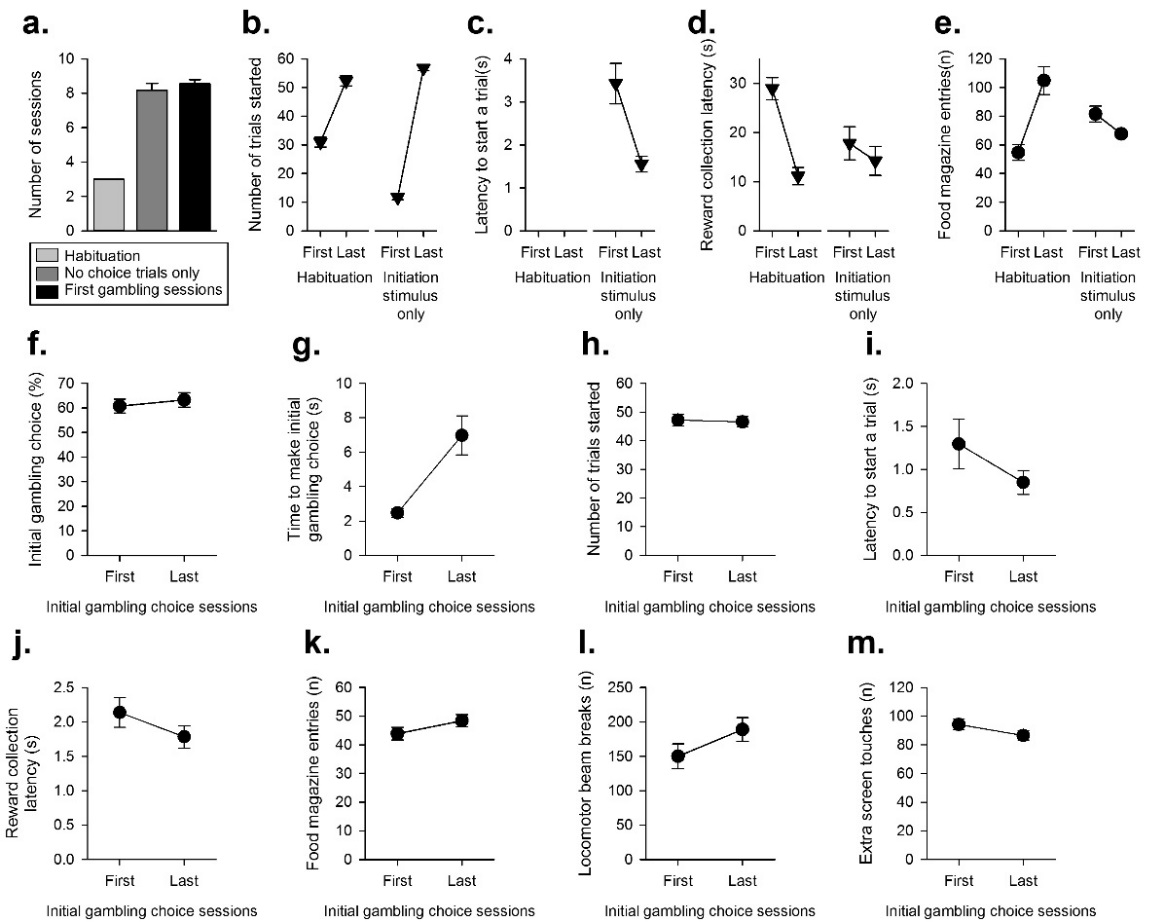 |
| --- |
| **Supplementary Results Figure 2:** Habituation and shaping the behaviour of adult C57Bl/6 mice. The mice showed demonstrable improvements in behaviour and performance of the task through the different stages of training (see main text and Supplementary Methods Figure 1): habituation and responding to the initiation stimulus only (b to e), and then performing a non-loss chasing version of the gambling task (G/LCT) (f to m). Mice were given 3 sessions to habituate to the chambers, but then worked independently to achieve criteria levels of stable behaviour in the next stages of training, taking on average ~8sessions/stage (a). During habituation, the mice successfully learnt that reward was present, as shown by increased trials (b) and food magazine entries (e) and a decrease in the latency to access the food magazine following reward delivery (d). Although trials initially decreased when the mice first had to touch the initiation stimulus to earn reward (b), their behaviour soon adapted, as demonstrated by increased numbers of trials completed and a speeding of their latency to touch the initiation stimulus (c). When on the GT the mice showed a preference to gamble, which had a 1:1 win/lose ratio, more than quit in their initial choice (f), slowing their choices (g) as their performance stabilised. Other task parameters remained relatively stable as the mice adapted to the demands of the GT (h to m). Data shows mean±SE, with both mouse cohorts combined (N=40). |

| 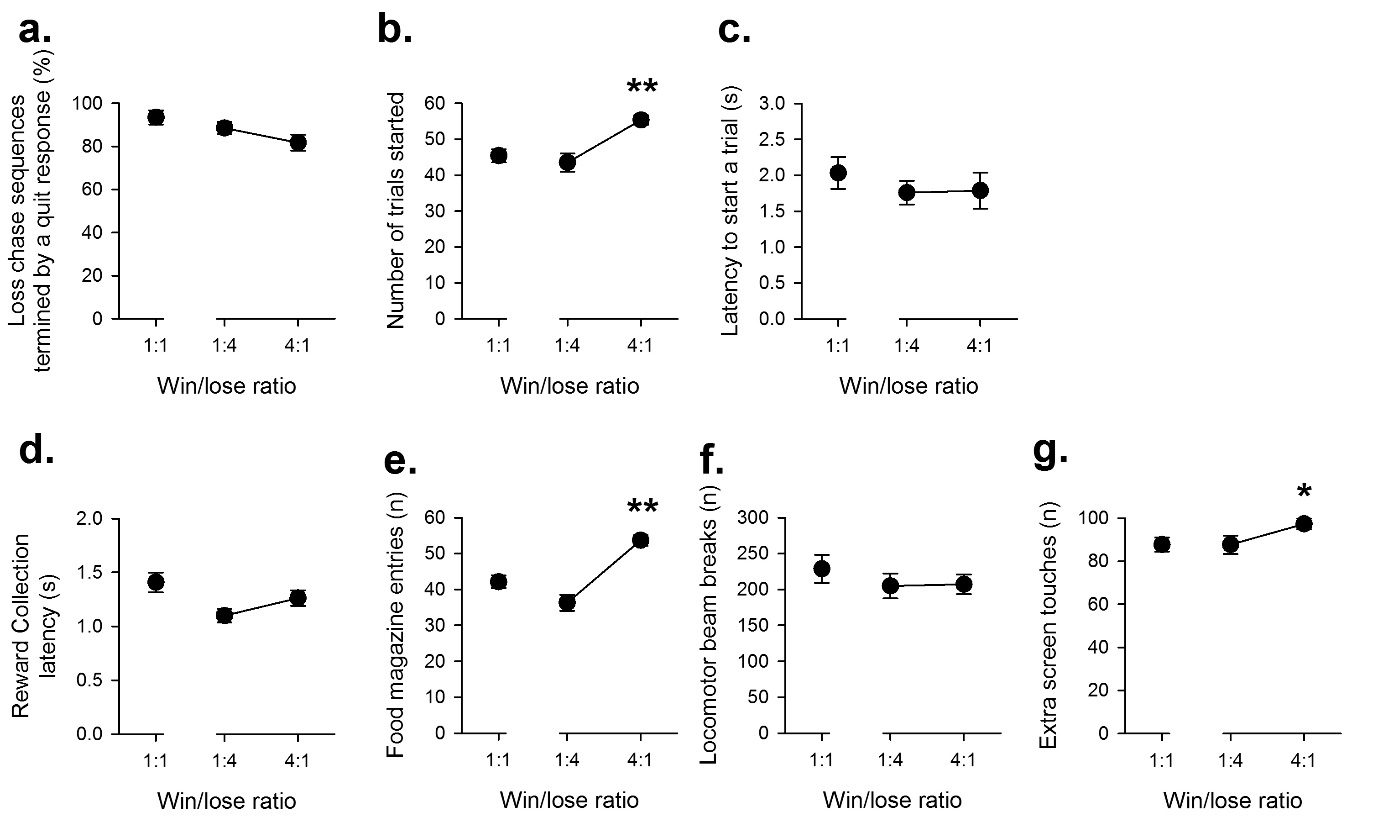 |
| --- |
| **Supplementary Results Figure 3:** Effects of altering the odds of winning on initial gambling choice and loss-chasing behaviour in both cohorts of adult C57Bl/6 mice: ancillary data. There were no effects of altering the win/lose ratio on how mice elected to end a loss-chase (a, t_39_=0.95, p=0.35), with the majority ended by the mice quitting. Mice started more trials in high win sessions (b, Z_39_=0.397, p=0.001), but there were no differences in the time taken to start a trial between high and low win sessions (c, Z_39_=0.82, p=0.41). There were no differences in the latency to collect reward (d, Z_39_=1.84, p=0.07), but the mice made many more entries into the food magazine when the odds of winning were greater (e, t_39_=5.09, p=0.001). Locomotor activity in these sessions was unaffected (f, t_39_=.11, p=0.91), but there was a significant increase in the amount of extra screen touches made by the mice in sessions with a 4:1 win/lose ratio (g, Z_39_=2.08, p=0.04). Data from the final day of testing at each schedule were used in the analysis. Data from sessions with a 1:1 win/lose ratio are shown for illustrative purposes and were not included in the statistical analysis. Data shows mean±SE, with both mouse cohorts combined (N=40). * denotes p<0.05 and ** denotes p<0.01 for the comparison between 4:1 and 1:4 win/ratio manipulations. |

| **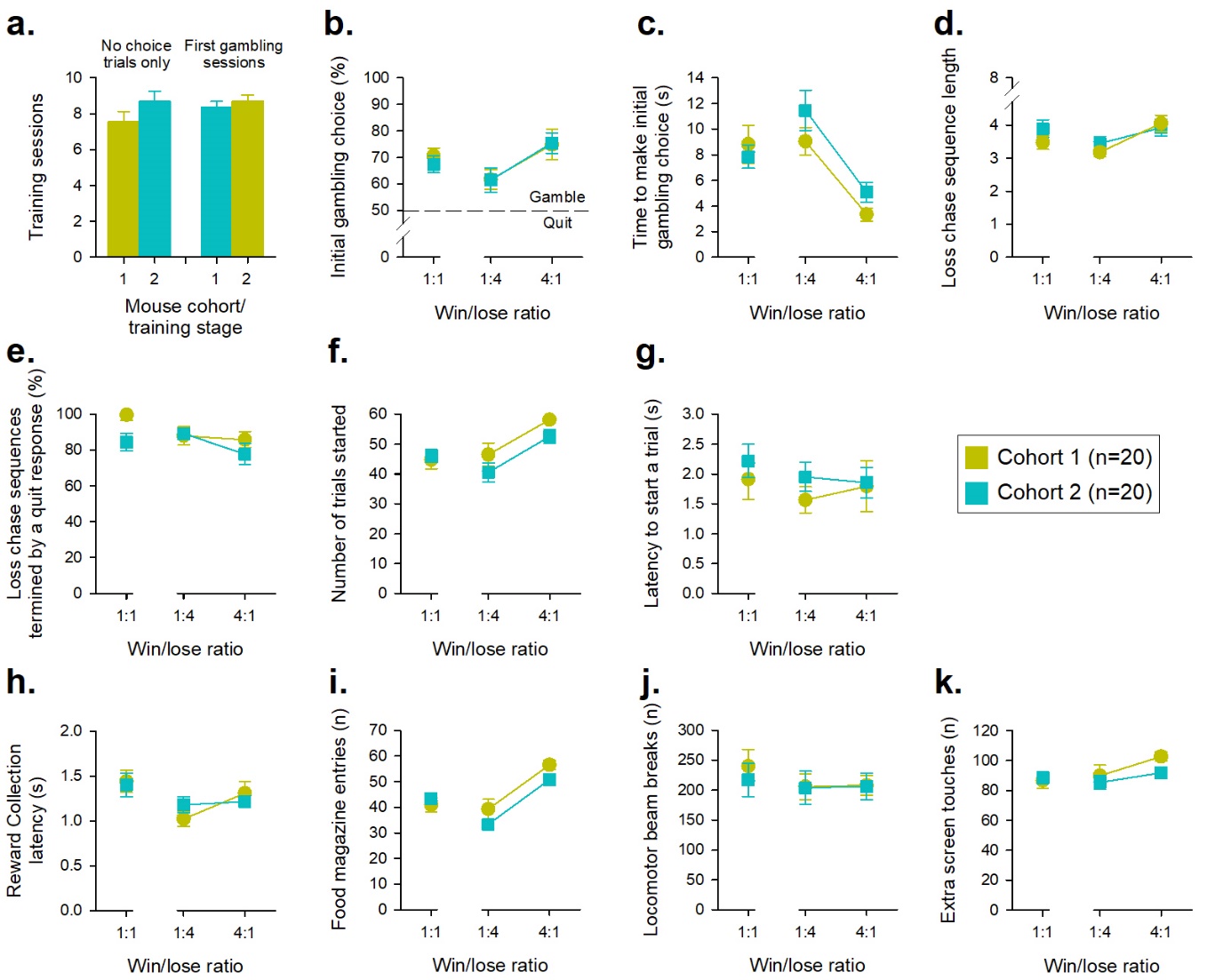** |
| --- |
| **Supplementary Results Figure 4:** Comparison of behaviour for both cohorts of C57Bl/6 mice used. As mentioned in the Materials and Methods, two cohorts of mice (N=20 in each) were used, and their data combined to generate overall performance measures for the task manipulations (shown in main text Figure 2). As can be seen from the graphs, there were no significant differences between the two groups of mice in terms of the number of training sessions taken to reach criteria in the responding to the initiation stimulus only, and then performing a non-loss chasing version of the gambling task (G/LCT) (a), or for the different parameters collected in sessions where the effects of altering the odds of winning on initial gambling choice and loss-chasing was evaluated (b to k). Furthermore, mice from each cohort demonstrated comparable alterations in behaviour following these task manipulations (main effect of COHORT, p>0.05, for all comparisons), confirmed by Bayesian analysis. Therefore, these data suggest that the separate cohorts of mice showed equivalent performance in the G/LCT, and that it was valid to combine the two sets of data for further analysis and investigation. Data from the final day of testing at each schedule were used in the analysis. Data from sessions with a 1:1 win/lose ratio are shown for illustrative purposes and were not included in the statistical analysis. Data shows mean±SE, N=20/cohort. |

| **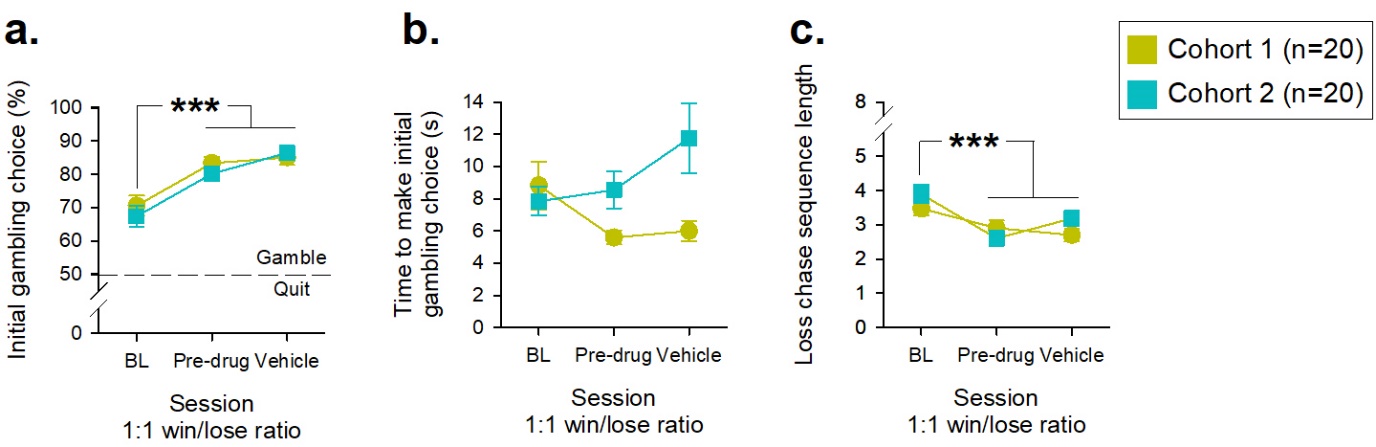** |
| --- |
| **Supplementary Results Figure 5:** Comparison of long-term performance of C57Bl/6 mice used. Following evaluation of the mice on the gambling task (G/LCT) with different win/lose ratio, sessions with a 1:1 win/lose ratio were reinstated ready for subsequent pharmacological investigations. There were some significant changes in behaviour in sessions with a 1:1 win/lose ratio between the initial baseline (BL) evaluations and after the task manipulations were completed, with increased initial gambling choices (a, main effect of SESSION, F_2,76_=25.87, p=0.001, η^2^=0.41) and reduced lengths of loss chase sequences (c, main effect of SESSION, F_2,76_=11.30, p=0.001, η^2^=0.23). Notably, however, these significant differences originated from differences between the first assessments of behaviour and the later pre-drug and on-drug (following vehicle administration) tests (*post-hoc* evaluations p<0.05); there were no differences in performance between pre-drug and vehicle treatment sessions (*post-hoc* evaluations p>0.05). This would suggest that although these measures changed with repeat testing, the performance of the mice reached an asymptote and remained constant during the evaluation of the pharmacological agents used. The time to make the initial decision to gamble did not differ with repeated testing (b, main effect of SESSION, F_2,76_=1.75, p=0.18, η^2^=0.04), and there were no significant differences between baseline, pre-drug and on-drug (following vehicle administration) tests (*post-hoc* evaluations p>0.05). Initial baseline, pre-drug and drug (following vehicle administration) comparisons of the other tasks parameters were not statistically different (main effect of SESSION, p>0.05 for all measures). Furthermore, there were no significant differences between the groups of mice for these or other task parameters (main effect of COHORT, p>0.05 for all measures), confirmed by Bayesian analysis. Data shows mean±SE, N=20/cohort. *** denotes p<0.001 for the comparison with baseline (BL) session. |
| 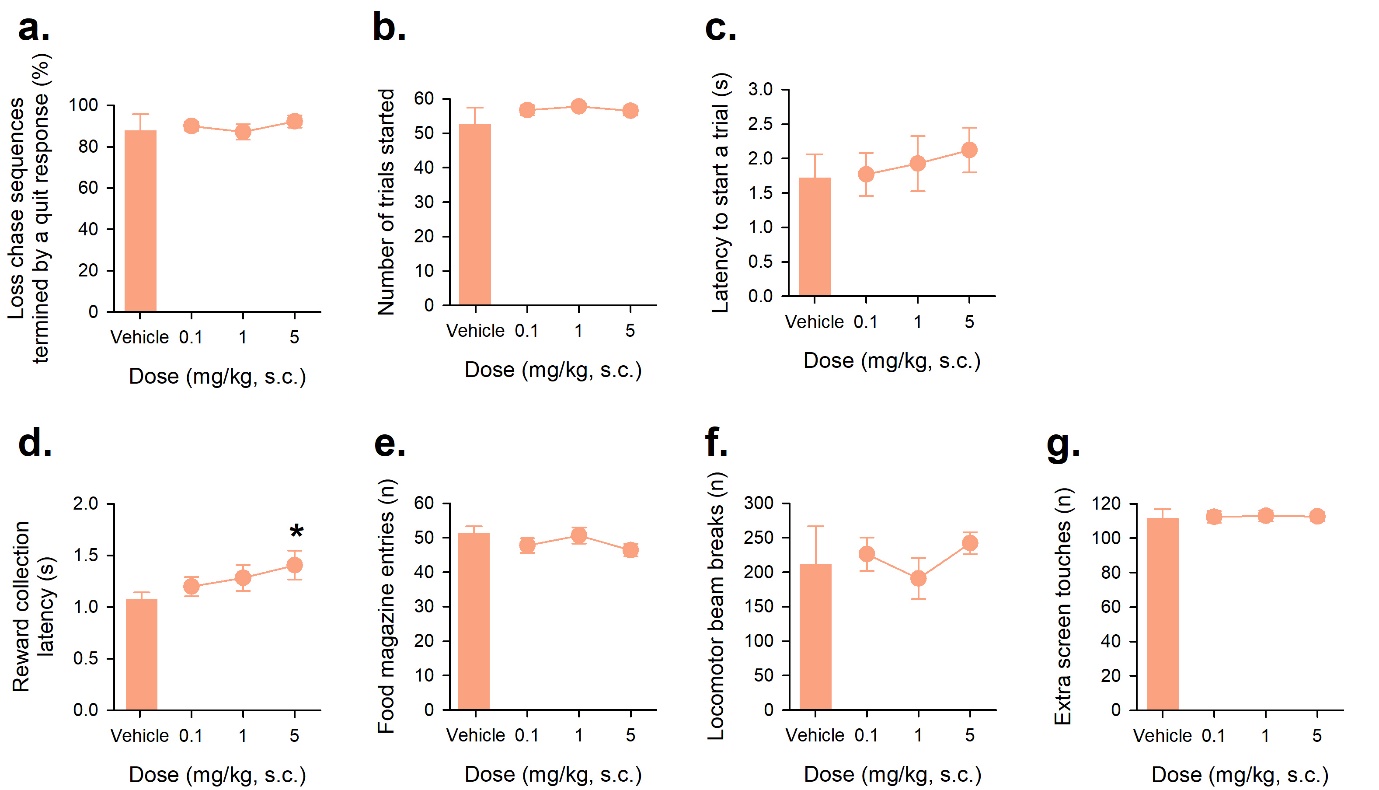 |
| **Supplementary Results Figure 6:** Effects of the 5-HT_2C_R antagonist SB242084 on initial gambling choice and loss-chasing behaviour in adult C57Bl/6 mice: ancillary data. SB242084 had little effect on the other parameters analysed including the number of loss-chases ended by quitting (a, main effect of DOSE, F_3,57_=0.87, p=0.46, η^2^=0.04), the number of trials completed (b, main effect of DOSE, F_3,57_=2.74, p=0.052, η^2^=0.13), the latency to start a trial (c, main effect of DOSE, F_3,57_=1.29, p=0.50, η^2^=0.03), and the numbers of magazine entries (e, main effect of DOSE, F_3,57_=2.10, p=0.11, η^2^=0.10), locomotor beam breaks (f, main effect of DOSE, F_3,57_=0.85, p=0.47, η^2^=0.04) and extra screen touches (g, main effect of DOSE, F_3,57_=0.01, p=0.99, η^2^=0.06). SB242084 did significantly increase the latency to collect the food reward (d, main effect of DOSE, F_3,57_=2.83, p=0.047, η^2^=0.13), although this was only a tendency for the highest dose used to differ from vehicle treatment as shown by *post hoc* pairwise comparison (p=0.076). For each of these sessions the win/lose ratio was 1:1. Data shows mean±SE, mice from Cohort 1 only (N=20). * denotes p<0.05 for the comparison with vehicle treatment. |

| 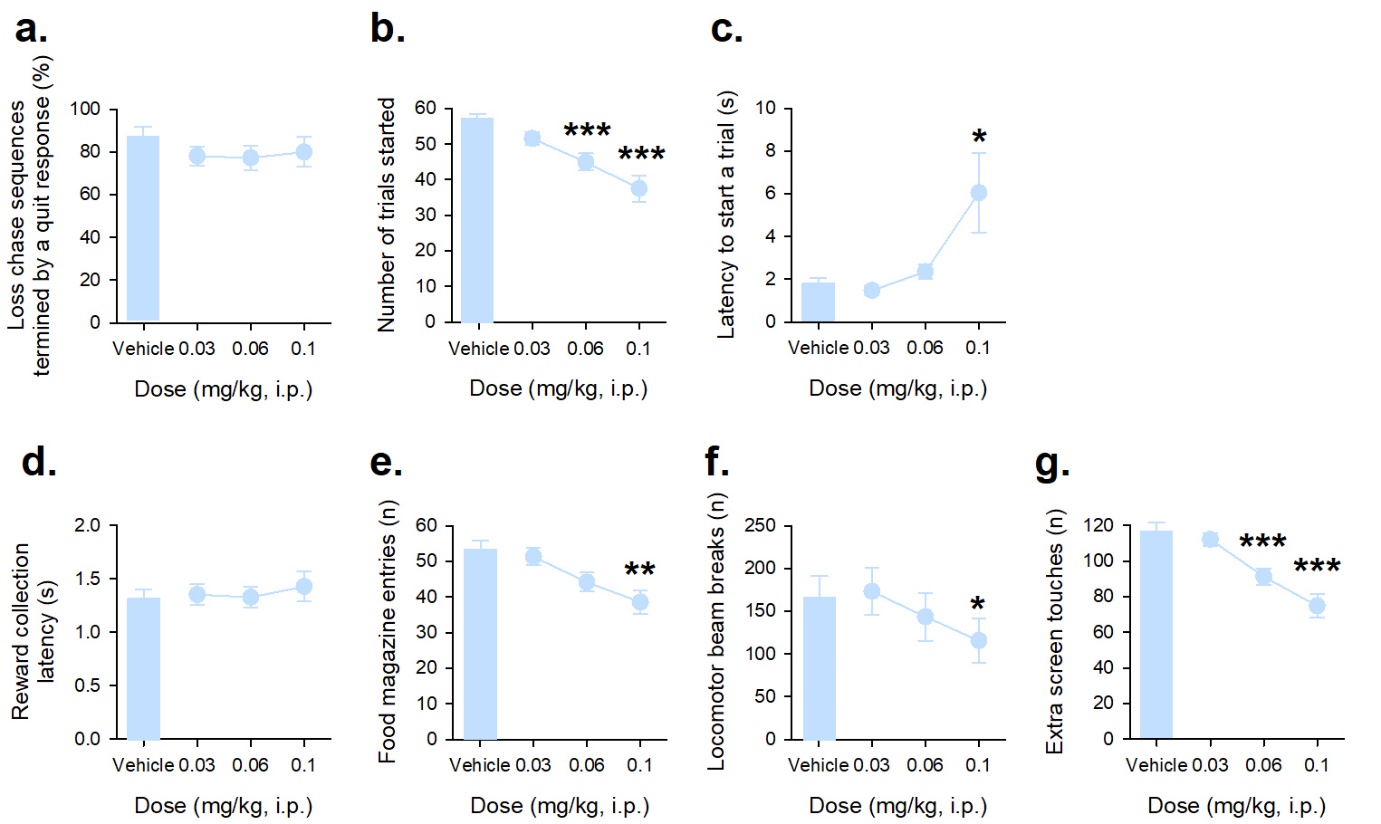 |
| --- |
| **Supplementary Results Figure 7:** Effects of the 5-HT_1A_R agonist 8-OH-DPAT on initial gambling choice and loss-chasing behaviour in adult C57Bl/6 mice: ancillary data. 8-OH-DPAT did not significantly affect how the mice ended loss-chasing sequences (a, main effect of DOSE, F_3,57_=0.75, p=0.53, η^2^=0.04), with the majority terminated by quitting. However, 8-OH-DPAT treatment led to significant dose-dependent reductions in the number of trials made (b, main effect of DOSE, F_3,57_=12.63, p=0.001, η^2^=1.00), a slowing of the latency to initiate a trial (c, main effect of DOSE, F_3,57_=10.80, p=0.001, η^2^=0.99), and significant decreases in the numbers of food magazine entries (e, main effect of DOSE, F_3,57_=8.15, p=0.001, η^2^=0.99), beam breaks (f, main effect of DOSE, F_3,57_=7.43, p=0.001, η^2^=0.98), extraneous screen touches (g, main effect of DOSE, F_3,57_=23.27, p=0.001, η^2^=1.00). *Post hoc* pairwise comparisons demonstrated that these significant effects occurred at the highest doses used (p<0.05) and that treatment with the 0.03mg/kg dose did not differ from vehicle for any measure. 8-OH-DPAT dosing did not affect the latency to collect the reward (d, main effect of DOSE, F_3,57_=0.29, p=0.84, η^2^=0.10). NB: Some mice (N=6) were treated with a 0.3mg/kg dose of 8-OH-DPAT but this led to major disruptions of behaviour and therefore this treatment was discontinued in the other subjects. For each of these sessions the win/lose ratio was 1:1. Data shows mean±SE, mice from Cohort 1 only (N=20). * denotes p<0.05, ** denotes p<0.01 and *** denotes p<0.001 for the comparison with vehicle treatment. |

| 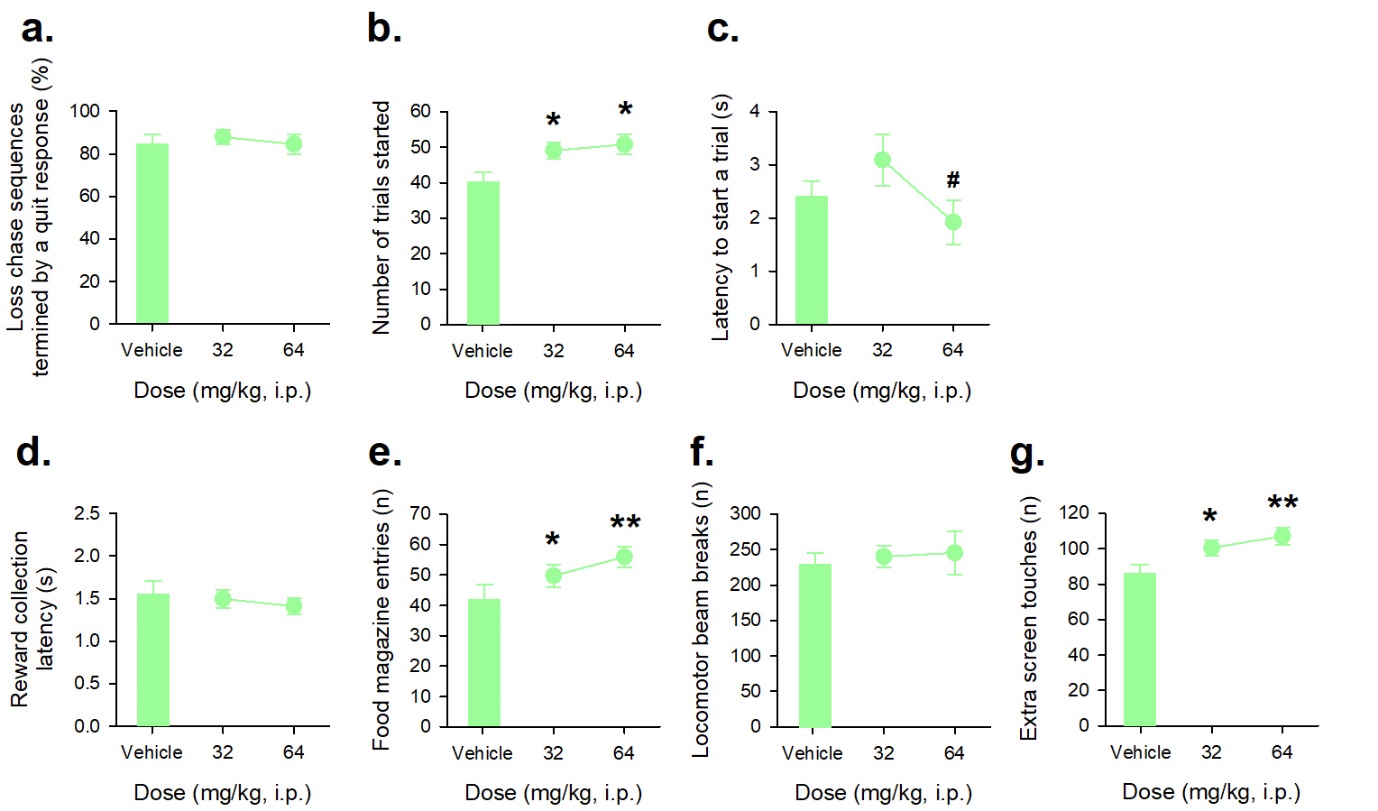 |
| --- |
| **Supplementary Results Figure 8:** Effects of modafinil on initial gambling choice and loss-chasing behaviour in adult C57Bl/6 mice ancillary data. Modafinil did not significantly affect how the mice ended loss-chasing sequences (a, main effect of DOSE, F_2,38_=0.18, p=0.83, η^2^=0.01), with the majority terminated by quitting. Increasing doses of modafinil significantly increased the numbers of trials started (b, main effect of DOSE, F_2,38_=7.14, p=0.002, η^2^=0.27), entries to the food magazine (e, main effect of DOSE, F_2,38_=8.57, p=0.001, η^2^=0.31) and extra screen touches (g, main effect of DOSE, F_2,38_=11.56, p=0.001, η^2^=0.38), There was also a significant effect of modafinil on the latency to initiate a trial (c, main effect of DOSE, F_2,38_=3.29, p=0.048, η^2^=0.15), p*ost hoc* pairwise comparisons showed neither the 32,g/kg or 64mg/kg treatments differed from vehicle (p>0.05), but that responding at 64mg/kg was quicker than following the 32mg/kg dose (p<0.05). Modafinil did not affect the latency to collect the reward (d, main effect of DOSE, F_2,38_=0.24, p=0.79, η^2^=0.01) or the amount of locomotor activity (f, main effect of DOSE, F_2,38_=0.19, p=0.83, η^2^=0.01). For each of these sessions the win/lose ratio was 1:1. Data shows mean±SE, mice from Cohort 2 only (N=20). * denotes p<0.05 and ** denotes p<0.01 for the comparison with vehicle treatment, and # denotes p<0.05 for the comparison between the 32mg/kg and 64mg/kg doses of modafinil. |
